# Cascaded Raman scattering based high power octave-spanning supercontinuum generation in graded-index multimode fibers

**DOI:** 10.1038/s41598-018-30252-9

**Published:** 2018-08-20

**Authors:** Uğur Teğin, Bülend Ortaç

**Affiliations:** 1 National Nanotechnology Research Center, Bilkent University, 06800 Bilkent, Ankara Turkey; 2 Institute of Materials Science and Nanotechnology, Bilkent University, 06800 Bilkent, Ankara Turkey

## Abstract

A new method to generate multi-watt-level, octave-spanning, spectrally flat supercontinua stemmed from cascaded Raman scattering in graded-index multimode fibers is reported. Formation dynamics of supercontinua are investigated by studying the effect of fiber length and core size. High power handling capacity of the graded-index multimode fibers is demonstrated by power scaling experiments. Pump pulse repetition rate is scaled from kHz to MHz while pump pulse peak power remains same and ~4 W supercontinuum is achieved with 2 MHz pump repetition rate. To the best of our knowledge, this is the highest average power and repetition supercontinuum source ever reported based on a graded-index multimode silica fiber. Spatial properties of the generated supercontinua are measured and Gaussian-like beam profiles obtained for different wavelength ranges. Numerical simulations are performed to investigate underlying nonlinear dynamics in details and well-aligned with experimental observations.

## Introduction

Supercontinuum generation in optical fibers is studied extensively in the past decades^1,2^. Today evolution dynamics of supercontinuum in single-mode or few-mode fibers are well understood. With the developments in photonic crystal fiber technology, fiber-based octave-spanning supercontinuum sources with wide range of parameters become feasible and nowadays fiber-based supercontinuum sources are widely used in variety of applications including optical metrology, fiber communication systems and biomedical imaging^3^. In recent years, graded-index multimode fibers (MMFs) drew huge attention by featuring unprecedented nonlinear phenomena. Nonlinear pulse propagation and modal interactions lead to discovery of unique phenomena such as Kerr self-cleaning^4,5^, spatio-temporal solitons^6^, ultrabroadband dispersive waves^7^, spatio-temporal instability^8–11^, quasi-phase matched intermodal four-wave mixing (FWM)^12^ and stimulated Raman scattering (SRS)^13,14^. With simultaneously exploiting these effects in graded-index MMFs various methods to generate supercontinua are presented in normal dispersion regime in the literature^15,16^.

First supercontinuum generation in graded-index MMF, reported by Lopez-Galmiche *et al*. with an enormous pump peak power such as 185 kW^15^. In this work Lopez-Galmiche *et al*. demonstrated the formation of supercontinuum from spatiotemporal instability, SRS and harmonic generation in 28.5 m graded-index MMF with 400 ps pulses at 1064 nm. Later Krupa *et al*. studied the generation of supercontinuum in graded-index MMF as well. Krupa *et al*. demonstrated the interplay between spatio-temporal instability and SRS while spatio-temporal instability peaks evolves to a supercontinuum^16^. These studies achieve supercontinuum generation by benefiting from the combinations of the complex nonlinear dynamics such as harmonic generation and spatiotemporal instability by choosing pump pulses with high peak power and low repetition rates such as ~50 mW with 500 Hz and ~700 mW with 30 kHz, respectively. Thus maximum average power of the reported octave-spanning supercontinua is in milliwatts range so far even though graded-index MMF is promoted with high power level handling potential. On the other hand, Chiang *et al*. demonstrated the generation of cascaded Raman scattering in graded-index MMF and studied the effect of excitation condition and the length of the test fiber on the spatial profile of the cascaded Raman Stokes peaks^13^. Later, Pourbeyram *et al*. employed the generation of cascaded Raman scattering in 1 km graded-index MMF and obtained 20 Raman peaks but these Raman peaks do not evolve to a continuum formation^14^.

In this paper, we report cascaded Raman scattering based novel method to generate octave-spanning high power and high repetition rate supercontinua in graded-index MMFs. We develop an all-fiber laser to obtain pump pulses with MHz repetition rates, ~30 kW peak power and 70 ps pulse duration at 1040 nm to excite the graded-index MMFs and spectrally flat octave-spanning supercontinua with multi-watt average output powers are generated. Formation and spectral broadening of supercontinuum are investigated numerically and experimentally. Variations of continuum spectra are reported with different fiber lengths and core sizes. Power handling capacity of the graded-index MMFs is demonstrated by scaling pump pulse repetition rate from 200 kHz to 2 MHz while peak power of the pump pulses remains same. While preserving supercontinuum spectrum, average output power is increased from 350 mW to ~4 W. Experimental studies revealed that spatial distribution of the obtained supercontinua in graded-index MMF features Gaussian-like beam shape. Although our experimental setup is pump power limited, our observations suggest higher average output powers can be achieved in graded-index MMFs with higher repetition rate pump laser systems.

## Results and Discussion

### Experimental Results

**Figure 1:**
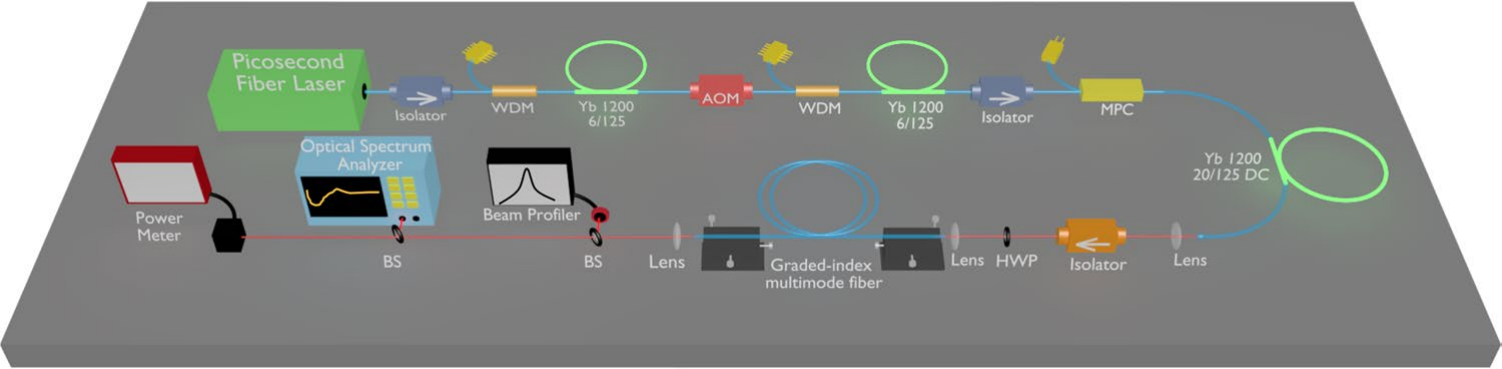
Schematic of the experimental setup comprising of Yb-doped fiber, wavelength division multiplier (WDM), beam splitter (BS), acousto-optic modulator (AOM), multi-pump combiner (MPC), three-dimensional stage (3DS), half-wave plate (HWP).

For different applications such as microscopy and frequency metrology, high average power supercontinuum sources are required to achieve adequate detection. All-fiber lasers are ideal candidates as pump sources to generate supercontinuum by offering high average and peak powers with compact size and perfect beam quality. On account of these advantages, we developed a home-built all-fiber laser as a source for supercontinuum generation experiments in graded-index MMFs. Figure 1 presents the schematic of the experimental setup. Yb-doped dispersion managed mode-locked fiber laser with 44 MHz repetition rate is employed as a pump pulse generator^17^. Generated pulses are chirped by a fiber stretcher to overcome possible nonlinear effects in amplification stages. Chirped pulses are first amplified by a preamplifier to ~70 mW average power. AOM is employed to decrease fundamental repetition rate of the pulse train before the main amplifier to increase peak power of the amplified pulses. To trigger cascaded Raman scattering in graded-index MMFs, kW peak powers with ps-ns pulse duration is required^14^. Due to the intrinsic losses of AOM and the change of repetition rate from 45 MHz to 200 kHz–2 MHz range, average power drops to <5 mW after the AOM. To prevent generation of amplified stimulated emission, another preamplifier is placed before the double clad main amplifier. At the end of the Yb-doped double clad main amplifier, we obtain 70 ps pulses with ~30 kW peak power centered around 1045 nm with ~20 nm bandwidth and adjustable repetition rate (kHz-MHz).

We collimate the amplified pump pulses with a biconvex lens and high power free-space isolator is used to prevent back reflections. Since the Raman gain is defined as polarization dependent^18^ we use a half-wave plate to change the polarization of the linearly polarized pump pulse. Here we aim to obtain best wavelength conversion condition to achieve supercontinua with wider spectral broadness in the graded-index MMF. To excite the fiber we prefer a biconvex lens with 2 cm focal length which creates ~20 *μ*m beam waist size at the fiber facet. Optimum coupling condition is ensured with a three-axis translation stage which also enables free space coupling efficiency greater than 80%.

**Figure 2:**
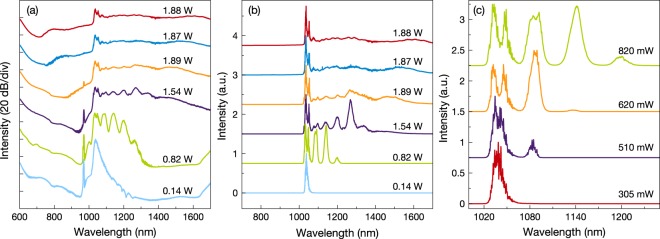
Variation of 20 m graded-index MMF as a function of launched pulse average power recorded for 1 MHz pump pulse repetition rate presented in (**a**) logarithmic and (**b**) linear scale. (**c**) Formation of cascaded SRS peaks. Output average powers indicated for each spectrum.

First, 20 m graded-index MMF with 62.5 *μ*m core diameter is selected as the test fiber and evolution of supercontinuum is investigated in detail by studying the effect of pump power [Fig. 2]. Starting from 510 mW output average power, generation of cascaded SRS with ~13 THz frequency shifts is observed as reported by Pourbeyram *et al*.^14^. Detailed generation of intense SRS peaks up to fifth Stokes is observed even in the linear scale [Fig. 2(b,c)]. This unique wavelength conversion mechanism based on Raman scattering can be explain with the multimode behavior of the fiber. As reported for the intermodal FWM^19^, generated Raman Stokes can also propagate in the higher order modes^14^. This propagation difference can compensate the velocity mismatch between the generated Raman Stokes and the pump pulse up to certain degree and lead to generation of the cascaded Raman scattering in graded-index MMFs. Before the average output power reaches to 1.89 W, when SRS peaks reach zero dispersion wavelength (ZDW) of the fiber (~1330 nm), cascaded Raman Stokes generation stops due to the reduction in SRS gain near ZDW^20^. Thus instead of a Raman Stokes, broad spectral formation emerges around 1500 nm. This formation is explained in the literature as complex parametric phenomena including collision based spectral broadening^2,21^.

**Figure 3:**
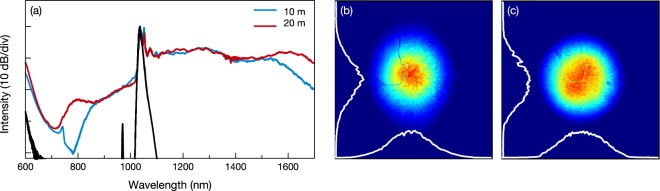
(**a**) 10 m and 20 m graded-index MMF for 1 MHz pump pulse repetition rate. Spectrum of pump pulse is presented as black. Near-field beam profile for (**b**) 730 nm to 1200 nm range and (**c**) 1100 nm to 1200 nm range of the supercontinuum.

After we reached 1.89 W output power we observed a small decrease at average output power and wavelength generation at shorter wavelengths starts to emerge. Fiber operation bandwidth is defined as 800 nm to 1600 nm thus attenuation of the fiber could be the reason of this power drop. The reason of evolution of supercontinuum at visible wavelengths can be explained with the coupling of Raman and parametric gain which also takes place in supercontinuum generation for picosecond pulses in photonic crystal fibers^22^. This results in the generation of anti-Stokes wavelengths even without proper phase-matching^23^. In the end, more than octave-spanning spectrally flat supercontinuum achieved with 1.88 W average output power for 1 MHz pump repetition rate. Overall spectral intensity deviation of the continuum is calculated as 52%. On the other hand, above the pump wavelength (between 1060–1700 nm), spectral intensity deviation decreases to 24%. Our observations suggest, spectral broadening triggered by the cascaded SRS peaks is the main reason of this outstanding spectral flatness. To understand the effect of the propagation length we decrease the fiber length to 10 m for the same conditions and its impact on the output average power and the spectral broadening is measured. The output average power increased from 1.88 W to 2.19 W due to decrease in the effective fiber loss to the propagating light. On the other hand, the spectral width and the flatness of the supercontinuum is decreased for the 10 m test fiber [Fig. 3(a)]. Similar behavior is also reported for various supercontinuum generation methods in different studies^16,22^. When we compare the results of Pourbeyram *et al*.^14^ with our experiments, our observations revealed wavelength of the pump pulse is an important parameter to generate supercontinuum in graded-index MMF and should not be far from the ZDW of the fiber. Since obtained results suggest strong soliton generation by wavelength generation above the ZDW of the fiber is essential to achieve supercontinua in graded-index MMFs. For the pump pulse central wavelengths far from the ZDW of the fiber, cascaded Raman scattering peaks do not evolve to a continuum^14^.

As a consequence of multimode behavior of the test fiber, the output beam quality of the supercontinuum is an interesting to study. Thus, we measure near field spatial distribution of the generated supercontinuum with a beam profiler. The beam profiler used in the experiments can operate up to 1200 nm. Due to the device limitation, we could measure the beam profile from 730 nm to 1200 nm Fig. 3(b). Additionally with a long-pass filter, we select the spectral content generated between 1100 nm and 1200 nm. Spatial distribution of the selected part of the supercontinuum is presented in Fig. 3(c). Even though fiber preferred in the experiments supports hundreds of modes, Gaussian-like spatial profile with high-order modes in the background is observed for both measurements. Similar spatial distributions are reported by previous studies on supercontinuum generation in GRIN multimode fibers^15,16^. Raman or Kerr beam cleaning could be the reason of observed spatial distributions^4,24^.

**Figure 4:**
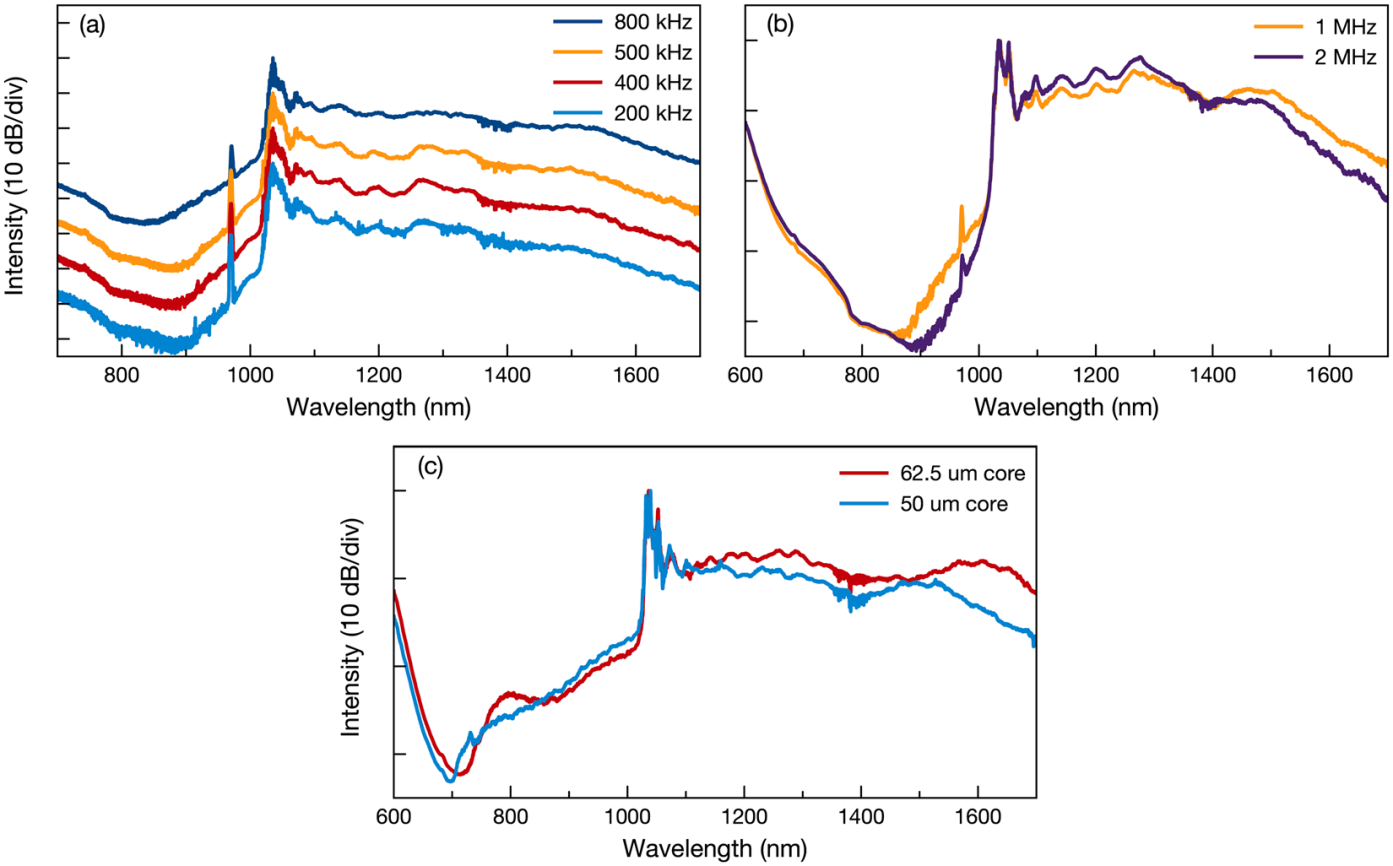
(**a**) Supercontinuum spectra measured from 20 m graded-index MMF (62.5 *μ*m core diameter) 200 kHz to 800 kHz repetition rates for constant peak power. (**b**) Supercontinuum spectra measured from 20 m graded-index MMF (62.5 *μ*m core diameter) for MHz repetition rates with same peak power. (**c**) Obtained supercontinuum spectra with 1 MHz pump pulses for graded-index MMFs with different core diameters.

To demonstrate the potential of the cascaded Raman scattering based supercontinuum generation method in graded-index MMF, while peak power of the pump pulse remains same, we change the repetition rate of the pump pulses from 200 kHz to 2 MHz. This allows us to scale average output power. First we focused on kHz range and set the pump peak power as 25 kW for 20 m test fiber [Fig. 4(a)]. The obtained average output powers are 350 mW, 700 mW, 875 mW and 1.4 W for 200 kHz, 400 kHz, 500 kHz and 800 kHz respectively. For the MHz repetition rates we compare the 1 MHz and 2 MHz separately due to pump power limitation we are facing at the main amplifier of our home-build fiber laser. As shown in Fig. 4(b), with increasing pump pulse repetition rate from 1 MHz to 2 MHz by preserving pump peak power ultra-broad supercontinua could be reproduced.

By doubling pump pulse repetition rate, we obtain 3.96 W and 3.50 W average output powers for supercontinua generated in 10 m and 20 m graded-index MMF with 2 MHz, respectively. To achieve these spectra we pump the graded-index MMF with 4.62 W pump pulses. With increasing the length of the test fiber, generating broader spectrum results in reduction at the average output power. Our scaling experiments are pump power limited but these results indicate that by increasing average power and repetition rate simultaneously, higher average powers can be obtained with standard graded-index fiber while octave-spanning supercontinuum features remain. Furthermore, we study supercontinuum generation in 20 m graded-index MMF with 50 *μ*m core diameter to demonstrate versatility of this low-cost supercontinuum generation technique. Even though self-imaging periods are different for 50 *μ*m and 62.5 *μ*m core diameters, similar cascaded Raman scattering and supercontinuum evolution also observed in graded-index MMF with 50 *μ*m core diameter. We believe the spatial propagation difference compensates the velocity mismatch between the generated Raman Stokes and the pump pulse to ensure generation of the cascaded Raman scattering also in the graded-index MMF with 50 *μ*m core diameter. For 1 MHz repetition rate and same average output power, spectral difference is presented in Fig. 4(c) with both fibers. The achieved supercontinuum features spectral resemblance with 62.5 *μ*m core diameter.

### Numerical Results

In order to develop better understanding on supercontinuum generation in graded-index MMFs, we perform numerical simulations. So far, researchers present various simulation techniques to study pulse propagation inside graded-index MMF^25–27^. These models are accurate and useful tools to understand spatiotemporal nonlinear effects in graded-index MMFs but simulations with relatively longer fiber lengths creates computational complexity and increases computation time. To overcome these problems additional to decreasing considered fiber modes in the simulations, computational tricks as decreasing pulse duration and fiber length generally preferred in the literature. These assumptions may be misleading when Raman process and shock terms are considered in the calculations. Recently, a fast modeling of pulse propagation inside graded-index MMF proposed to overcome these issues^7,28^. This model is based on 1 + 1D generalized nonlinear Schrödinger equation (Eq. 1) with a periodic nonlinear coefficient *γ*(*z*) to imitate spatiotemporal beam propagation inside the graded-index MMFs.

1 $$\frac{\partial A}{\partial z}+(\sum _{n\ge 2}\,{\beta }_{n}\frac{{i}^{n-1}}{n!}\frac{{\partial }^{n}}{\partial {t}^{n}})A=i\gamma (z)(1+\frac{\partial }{\partial t})(\mathrm{(1}-{f}_{R})A{|A|}^{2}+{f}_{R}A{\int }_{0}^{\infty }{h}_{R}(t^{\prime} ){|A(z,t-t^{\prime} )|}^{2})$$

2 $${A}_{eff}(z)=2\pi {a}_{0}^{2}[co{s}^{2}(\sqrt{g}z)+\frac{1}{{\beta }_{0}^{2}{a}_{0}^{4}g}si{n}^{2}(\sqrt{g}z)]$$

Self-imaging pattern is implemented to nonlinear coefficient as *γ*(*z*) = (*ω*_0_*n*_2_)/(*cA*_*eff*_(*z*)) where *ω*_0_ is central frequency, *c* is speed of the light and *A*_*eff*_(*z*) is effective area defined as Eq. 2. In Eq. 2, $g=2{\rm{\Delta }}/{r}_{c}^{2}$ where *r*_*c*_ is fiber core radius, ${\rm{\Delta }}=({n}_{core}^{2}-{n}_{clad}^{2})\mathrm{/2}{n}_{core}^{2}$ is the relative index difference between the core and the clad of the fiber and *β*_0_ = *ω*_0_*n*_0_/*c* where *n*_0_ is the core refractive index (at the center of the fiber).

**Figure 5:**
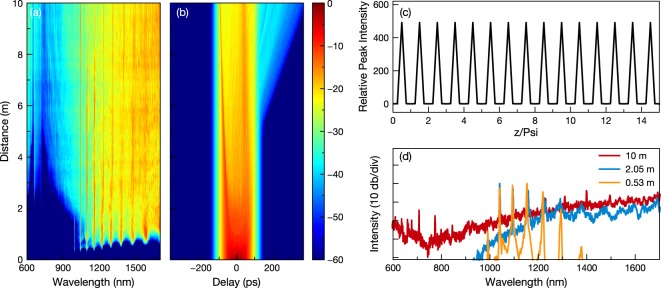
Results obtained by averaging of numerical simulations showing (**a**) spectral and (**b**) temporal evolution through 10 m graded-index MMF with 62.5 *μ*m core diameter. (**c**) Relative peak intensity imposed by nonlinear coefficient in simulations. Self-imaging period Psi = 503.6 *μ*m. (**d**) Numerically obtained spectral evolution for different propagation lengths.

Spectral and temporal evolutions obtained from numerical simulations for 10 m graded-index MMF with 62.5 *μ*m core diameter are presented in Fig. 5(a,b). Pump pulse is defined as 70 ps duration with 30 kW peak power and centered at 1040 nm. For numerical integration with high accuracy, we prefer the fourth-order Runge-Kutta in the Interaction Picture method^29,30^. We set beam spot size at the fiber facet (*a*_0_) as 20 *μ*m, *n*_2_ as 3.2 × 10^−20^ *m*^2^/*W*, relative index difference as 0.019, time window width as 750 ps with 2 fs resolution. In simulations, we include Raman process (*f*_*R*_), shock terms and high-order dispersion coefficients up to *β*_7_. SRS is included in the equation via use of a response function^31^. Even though our simulations start from quantum noise, we average the simulations over 4 sets of initial conditions to simulate experimental observations more accurately. Relative peak intensity ${a}_{0}^{2}/{a}^{2}(z)$ obtained with aforementioned parameters is presented in Fig. 5(c). To avoid aliasing in periodic nonlinearity term, integration step is defined as 125.91 *μ*m.

Evolution of the supercontinuum also contains cascaded SRS generation in the numerical calculations. To indicate spectral broadening and wavelength conversion, spectra after 50 cm, 2 m and 10 m propagation are presented in Fig. 5(d). After SRS peaks reach to ZDW, generation of new wavelengths at anomalous dispersion resembles development of Raman soliton components. In the literature, this phenomenon is explained as the transformation of SRS peaks above ZDW to ultrashort solitons^32^. These solitons experience self-phase modulation and more uniform spectra can be formed. Preceding soliton dynamics lead to temporal breakup seen in the simulations after 5 m propagation and with the help of spectrally broadened SRS peaks. Even though loss terms are not included in the simulations, spectral flatness and intensity distribution behavior matches well with experiments.

## Conclusion

In summary, we report a novel technique to generate high average power spectrally flat octave-spanning supercontinua triggered by cascaded Raman scattering using a graded-index MMF pumped with an all-fiber laser system. The highest supercontinuum output power of 3.96 W is achieved in graded-index MMF with 62.5 *μ*m core diameter using picosecond pulses at MHz repetition rate. Experimental and numerical studies reveal that unique cascaded SRS observed in graded-index MMF plays a significant role in the octave-spanning spectral evolution. Spatial distribution of the generated supercontinua obtained for different wavelength ranges and Gaussian-like beam shape is measured. We have shown that this low-cost graded-index multimode fiber-based supercontinuum source could benefit from multimode features of the fiber and high power level average powers are feasible to achieve. Adaptability of the high power and high repetition rate supercontinuum generation method is studied as well. Further power scaling based on fiber pump lasers enables low-cost high repetition rate all-fiber supercontinuum systems with >10 W average powers.

## Methods

The experimental setup presented in Fig. 1. Yb-doped active fibers (nLight) are used in the oscillator and the amplifiers. Thorlabs GIF50C and GIF625 graded-index MMFs are used in the experiments. Pump pulse repetition rate is adjusted via AOM (AA Opto-Electronics MT200-IR10). The spectral measurements are recorded with Yokogawa AQ6370D spectrum analyzer. Spatial distribution of the generated supercontinua is obtained by DataRay (WinCamD-UCD23) beam profiler.
